# Household Water Quantity and Health: A Systematic Review

**DOI:** 10.3390/ijerph120605954

**Published:** 2015-05-28

**Authors:** Rachel D. Stelmach, Thomas Clasen

**Affiliations:** 1 Department of Global Health, Rollins School of Public Health, Emory University, Atlanta, GA 30322, USA; E-Mail: rstelma@emory.edu; 2 Department of Environmental Health, Rollins School of Public Health, Emory University, Atlanta, GA 30322, USA

**Keywords:** water supply, water quantity, diarrhea, trachoma

## Abstract

While the quantity of water used in the home is thought to be an important determinant of health, much of the evidence relies on using water access as a proxy for quantity. This review examines the health effects of household water quantity using studies that directly measured water quantity. We searched MEDLINE, EMBASE, the Cochrane Library, Web of Science, and article reference lists. Eligible studies included experimental and observational studies that measured a difference in water quantity and quantified an association between water quantity and health outcomes. 21 studies, divided into six of the many possible water-quantity associated outcomes, met the eligibility criteria. Due to heterogeneity in designs, settings, methods, and outcomes, a meta-analysis was inappropriate. Overall results showed a positive association between water quantity and health outcomes, but the effect depended on how the water was used. Increased water usage for personal hygiene was generally associated with improved trachoma outcomes, while increased water consumption was generally associated with reduced gastrointestinal infection and diarrheal disease and improved growth outcomes. In high-income countries, increased water consumption was associated with higher rates of renal cell carcinoma and bladder cancer but not associated with type II diabetes, cardiac-related mortality, or all-cause mortality.

## 1. Introduction

International efforts related to water in low-income countries often focus on improving water supplies. The WHO/UNICEF Joint Monitoring Program for Water and Sanitation (JMP), which is charged with monitoring progress toward Millennium Development Goal (MDG) 7, counts water supplies as “improved” based on the level of service. Piped water, public taps or standposts, tubewells or boreholes, protected dug wells, protected springs, and rainwater are considered “improved”; unprotected dug wells, unprotected springs, and surface water are deemed “unimproved”. Significantly, the distinction between improved and unimproved supplies is based principally on the quality of water they produce: their perceived, though often untested, potential to deliver safe drinking water sustainably [1]. Water quantity is not directly part of the current criteria for designating improved water supplies.

This lack of focus on water quantity also appears in much of the epidemiological literature. In recent years, numerous systematic reviews have assessed the impact of water on health, especially diarrheal diseases [2,3,4,5,6,7,8]. Other reviews have examined the impact of water on nutritional status [9], soil-transmitted helminth infections [10] and trachoma [11,12]. These reviews, however, either focus on “improved” *versus* “unimproved” water supplies, on specific types of water supplies (e.g., piped water), or on improvements in water quality. Only Esrey and colleagues aimed to assess the health impact of improvements in water quantity independently from water quality [13,14]. While they reported water quantity interventions to be protective against diarrheal disease, most of the underlying studies did not measure water quantity directly. Instead, they used distance to water supplies as a proxy for quantity [15]. The reviews, which now date back nearly a quarter century, also rely heavily on studies with before/after comparisons and other methodological shortcomings.

Despite the paucity of evidence on the health impact of water quantity, there are a number of recommendations related to minimum levels of water in the home. The SPHERE project sets out 15 litres (L) of water used *per capita* per day as a minimum standard for disaster relief [16]. A guidance document prepared for the UK Department of International Development (DFID) suggested that a minimum criterion for water supply should be 20 L *per capita* per day (lpcd) [17]. The same figure has been suggested by other researchers [18]. Gleick suggested that the international community adopt a figure of 50 lpcd as a basic water requirement for domestic water supply [19]. The WHO has not yet issued guidelines on water quantity, as it has for decades on water quality [20]. Nevertheless, in a widely quoted background document, the WHO cites a minimum for basic health protection of at least 20 L per person per day, of which 7.5 L is required for consumption, including direct hydration and cooking [21].

This review summarizes evidence on the impact of improvements in water quantity at the household level. It examines studies in any population in any region of the world. It should be noted that this review does not include studies where interventions address water supplies and in doing so, might increase the amount of water available in the home. In order to be included in this review, studies must have actually measured a difference in water quantity at the household level, not a proxy such as improved water supplies, improved access, improved storage, or reduced time to collect water.

## 2. Methods

### 2.1. Criteria for Selecting Studies

Study eligibility was determined based on the study design, the exposure of interest, the outcome measures, and the reported measures of effect.

- Eligible study designs included randomized controlled trials (RCTs); non-randomized studies (NRS) with a control group, including quasi-RCTs, non-randomized controlled trials, and controlled before-and-after studies; interrupted time-series studies; historically controlled studies; case-control studies; cohort studies; and cross-sectional studies.
- The exposure of interest was a measured change or difference in the quantity of water used in the home. Self-reported or estimated measures of water quantity were acceptable, but proxy measures such as distance to a water source or number of contacts with a water source were ineligible.
- The outcomes of interest were direct health outcomes measured at the individual or household level. Intermediate health outcomes such as cellular or metabolic processes were excluded.
- There had to be at least one quantified measure of effect linking water quantity and the health outcome of interest.

Eligible languages for inclusion were English, French, Spanish, Portuguese, and Italian. Only published, peer-reviewed records were considered. There were no restrictions on date or location.

### 2.2. Search Methods

The databases searched were the Cochrane Library—which includes the Cochrane Central Register of Controlled Trials (CENTRAL), the NHS Economic Evaluation Database, and the Cochrane Database of Systematic Reviews—EMBASE, MEDLINE via OVID, and Web of Science. First, the databases were searched for articles containing references to water supply or to interventions that could potentially change the quantity of water used; this search was further restricted by requiring references to a volumetric measure of water or to a phrase similar to “water quantity.” In a separate search, the databases were searched for a list of health outcomes of interest; these were then combined to create a list of articles referencing the outcomes of interest. Finally, the exposure and outcome searches were combined to create the initial list of articles. Please see supplemental file Table S1 for a detailed description of the search terms used for each database. In addition, the bibliography of the 1991 review by Esrey *et al.* was examined for additional relevant records [14].

### 2.3. Data Collection, Extraction, and Analysis

We used the above search strategy to compile an initial list of records. We then screened the titles and abstracts of the records based on the inclusion criteria. The full texts of all records that either appeared relevant or could not be rejected with certainty were then obtained. Two reviewers independently examined the full text of these records for final inclusion; all records excluded at this stage were recorded with reasons for exclusion. The notes on reasons for exclusion were then used to determine inclusion when the reviewers disagreed.

Data on study population, study methods, water quantity measures, and health outcomes were extracted using a standardized form. If no odds ratios and/or risk ratios were given but the authors provided sufficient raw data, we calculated the appropriate measure of effect.

### 2.4. Risk of Bias

The screening of studies was based on the WHO GRADE approach, with procedures as described in Strunz *et al.* [10]. Studies were assessed for risk of bias on five criteria: measurement of exposure, measurement of outcome, control for potential confounding, response rate, and selective reporting. For the measurements, self-report without researcher confirmation was noted as high risk, while measurements directly confirmed through observation by the researchers were noted as low risk. If the measures of effect were adjusted through statistical analysis or study design, they were marked as low risk; if no adjustment occurred, this category was marked as high risk. Response rates of above 80% were marked as low risk, while those below 80% were marked as high risk; if no information was given on response rate, this category was marked as unclear. The risk of selective reporting was deemed high if the studies named comparisons for which they did not provide measures of effect, especially if these comparisons were deemed not statistically significant with no other data given. An overall risk of bias was then compiled based on these categories.

## 3. Results

### 3.1. Description of Studies

**Figure 1:**
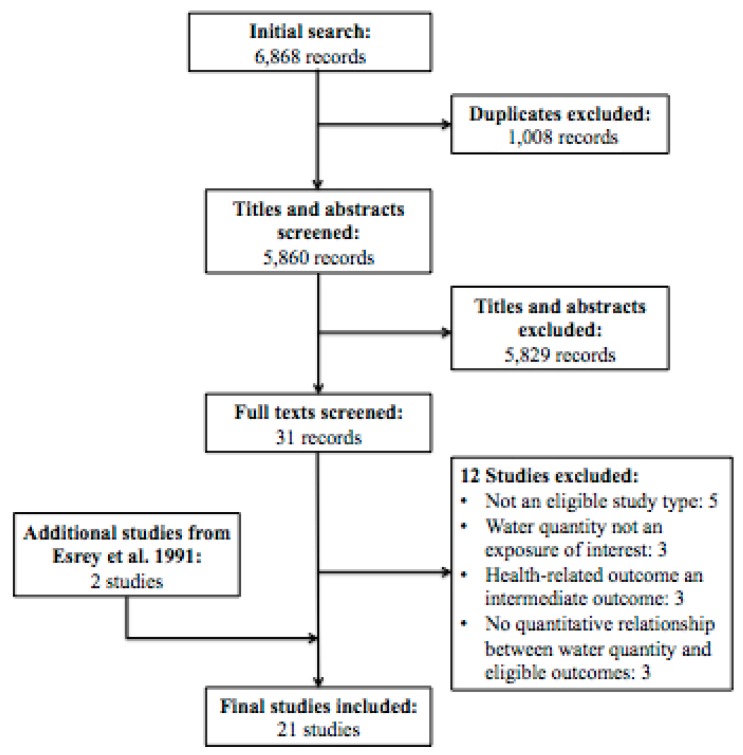
Study Flow Diagram.

As shown in Figure 1, of the 6,868 unique records screened, 19 were selected for final inclusion in the review. Two additional studies were identified through the bibliography of the Esrey 1991 review [14], yielding a total of 21 unique studies that met the review’s eligibility criteria. The studies took place in 14 countries, of which three are high-income, five are upper-middle or lower-middle income, and six are low-income as of 2014 [22]. All included records were in English.

### 3.2. Risk of Bias

Fifteen of the 21 included studies were cross-sectional studies. The overall risk of bias for the included studies was generally high (Table 1).

**Table 1:** Assessment of bias

Study	Study Type	Exposure Measure	Outcome Measure	Control for Confounding	Response Rate	Selective Reporting	Overall Risk of Bias
Aggarwal 2012 [23]	Retrospective cohort	unclear	unclear	low	low	low	**high**
Bailey 1991 [24]	Case control	low	low	low	low	low	**low**
Cairncross 1987 [25]	Cross-sectional	low	high	high	unclear	high	**high**
Esrey 1989 [26]	Cross-sectional	low	high	low	unclear	low	**high**
Esrey 1992 [27]	Cross-sectional	high	low	low	low	low	**high**
Hebert 1985 [28]	Cross-sectional	high	low	low	low	low	**high**
Hu 2009 [29]	Case control	high	low	low	high	low	**high**
Jones 2007 [30]	Cross-sectional	high	high	low	high	high	**high**
Khan 1982 [31]	Cross-sectional	low	low	high	unclear	high	**high**
Kupka 1968 [32]	Cross-sectional	low	low	high	unclear	low	**high**
Mahande 2012 [33]	Case control	high	low	low	unclear	low	**high**
Moalic 2000 [34]	Cross-sectional	high	low	high	unclear	low	**high**
Palmer 2012 [35]	Prospective cohort	high	low	low	low	low	**low**
Pan 2012 [36]	Prospective cohort	high	low	low	low	low	**low**
Polack 2006 [37]	Cross-sectional	low	low	low	low	low	**low**
Shrestha 2013 [38]	Cross-sectional	high	high	low	low	low	**high**
Teklemariam 2000 [39]	Cross-sectional	high	high	high	low	low	**high**
Tumwine 2002 [40]	Cross-sectional	low	high	low	unclear	low	**low**
Vena 1993 [41]	Case control	high	low	low	unclear	high	**high**
West 1989 [42]	Cross-sectional	high	low	low	unclear	low	**high**
Zhang 2013 [43]	Cross-sectional	low	low	high	unclear	high	**high**

### 3.3. Effects of Intervention

#### 3.3.1. Trachoma

Seven studies, shown in Table 2, examined the relationship between trachoma and water quantity. Four were cross-sectional studies with a high risk of bias [25,32,34,42], one was a case-control study with a high risk of bias [33], one was a cross-sectional study with a low risk of bias [37], and one was a case-control study with a low risk of bias [24]. Four studies specifically examined trachoma in rural children [24,33,34,37], with three further specifying age ranges of 1–6 years old [33], 1–9 years old [37], and less than 15 years old [24]. The other three studies examined trachoma in rural households as a whole [25,32,33], with one of those further specifying that the households had to include at least two children between 1 and 9 years of age [33].

Trachoma indicators were not found to be significantly associated with the amount of water brought to the house in three of the five studies using that measure [24,37,42], although one study found a significant protective association [33] and one noted a comparison without providing statistical analysis [25]. Two studies measured the quantity of water used for all domestic tasks. One found no association between trachoma and water usage [37], and the other found an association between water usage and active trachoma only among children aged 1–15 years old [32]. Evidence of the association of trachoma with water quantity usage for bathing children was mixed, with one study finding a significant effect and another a lack of significant effect [24,34]. In contrast, in the two studies that examined it, the quantity of water used in face washing—particularly face washing for children—was significantly associated with better trachoma outcomes [37,33].

#### 3.3.2. Gastrointestinal Illness

Six studies, shown in Table 3, examined the relationship between water quantity and gastrointestinal (GI) illness, with specific outcomes including acute gastrointestinal illness, diarrheal disease, shigellosis infections, and *Giardia lambia* infections. All of the studies were cross-sectional studies, with five having a high risk of bias [26,30,31,38,39] and only one having a low risk of bias [40]. Two studies examined GI illness in children [26,39], with one of these further specifying an age range of 1–5 years old [39]. The other four studies provided no age restrictions on their participants [30,31,38,40].

In the two studies that examined household water usage, increased usage was found to be significantly associated with decreased incidence of GI illness, specifically diarrheal disease and *Giardia lambia* infections [26,40]. Another study, which examined cooking and drinking water usage separately from bathing and washing water usage, found a significant relationship between shigellosis infection and water usage for bathing and washing, but only for those whose family contacts received a hand washing training intervention [31]. The amount of water consumed, however, was found in two studies not to have an association with the incidence of diarrheal illness [38,39], and in one study, higher amounts of water consumed were actually associated with an increased incidence of acute GI illness [30].

#### 3.3.3. Growth Indicators

Three studies, shown in Table 4, examined growth indicators, including height, weight, and combinations of those two indicators. All three of the studies were cross-sectional studies with a high risk of bias. All three of the studies focused on children, with one including infants aged between 0 and 12 months [27], one including children between the ages of 0 and 6 years old [28], and one including children between 8 and 17 years old [43].

**Table 2:** Effects of Water Quantity on Trachoma Outcomes.

Study	Setting and Participants	Trachoma Indicator	Water Quantity Measure(s)	Measure of Effect	Comparison Groups *	Values *	*p* Value
Bailey 1991 [24]	Gambia; rural children, <15 years	Household (HH) with at least one active trachoma case	Water brought to house	Comparison	Trachoma *vs.* no trachoma	15.8 *vs.* 17.9 liters (L) per HH per day	>0.05
			Water usage for bathing children	Comparison	Trachoma *vs.* no trachoma	4.2 *vs.* 6.4 L per child per day	0.03
Cairncross 1987 [25]	Mozambique; residents of rural HH	Prevalence of trachoma	Water brought to house	Comparison	High prevalence *vs.* low prevalence	8 *vs.* 14 liters per capita per day (lpcd)	unclear
Kupka 1968 [32]	Morocco; residents of rural HH	Active trachoma (among <1 year olds)	Water usage	Odds ratio (OR)	<5 lpcd	(ref)	
					5–10 lpcd	0.71 (0.13, 3.33)	0.69
					>10 lpcd	undefined	undefined
		Active trachoma (among 1–15 year olds)	Water usage	OR	<5 lpcd	(ref)	
					5–10 lpcd	0.79 (0.38, 1.54)	0.51
					>10 lpcd	0.34 (0.14, 0.82)	0.01
		Active trachoma (among >15 year olds)	Water usage	OR	< 5 lpcd	(ref)	
					5–10 lpcd	0.89 (0.61, 1.31)	0.63
					>10 lpcd	0.99 (0.60, 1.63)	0.96
		Severe trachoma (all ages)	Water usage	OR	<5 lpcd	(ref)	
					5–10 lpcd	0.93 (0.68, 1.27)	0.63
					> 10 lpcd	1.01 (0.64, 1.58)	0.96
Mahande 2012 [33]	Tanzania; residents of rural HH with at least 2 children 1–9 years	HH with at least 2 children with active trachoma	Water brought to house	OR	≥60 L *vs.* <60 L	0.40 (0.10, 0.30)	<0.001
			Water usage for face washing	OR	≥2 L *vs.* 1 L	0.01 (0.02, 0.07)	<0.001
			Water usage for bathing children	OR	>20 L *vs.* 10–20 L	0.90 (0.24, 0.80)	>0.05
Moalic 2000 [34]	Senegal; rural children (no age)	Child with trachoma	Water usage for washing	Comparison	Trachoma *vs.* no trachoma	8.6 L *vs.* 9.3 L	0.04
Polack 2006 [37]	Tanzania; rural children, 1–9 years	Child with trachoma	Water brought to house	OR	≤8 lpcd	(ref)	>0.05
					9–15 lpcd	1.02 (0.53, 1.94)	
					15–20 lpcd	1.12 (0.51, 2.47)	
					>20 lpcd	1.22 (0.68, 2.18)	
			Water usage	OR	3.8–11.3 lpcd	(ref)	>0.05
					11.3–14.6 lpcd	0.93 (0.27, 3.24)	
					14.6–21.3 lpcd	0.81 (0.23, 2.88)	
					3.8–11.3 lpcd	1.04 (0.26, 3.39)	
			Water usage for face washing	OR	<2 lpcd	(ref)	<0.05
					2–3.7 lpcd	0.32 (0.10, 1.06)	
					3.8–5 lpcd	0.08 (0.02, 0.31)	
					>5 lpcd	0.05 (0.01, 0.25)	
			Water usage for face washing children	OR	<2 lpcd	(ref)	<0.01
					2–3.5 lpcd	0.39 (0.11, 1.34)	
					3.6–5 lpcd	0.29 (0.08. 1.11)	
					>5 lpcd	0.33 (0.09, 1.17)	
West 1989 [42]	Tanzania; rural children, 1–6 years	HH with at least one active trachoma case	Water brought to house	OR	<25 L	(ref)	>0.05
					25–45 L	1.01 (0.76, 1.35)	
					>45 L	0.84 (0.61, 1.15)	

* All comparisons are in units of water per day.

**Table 3:** Effects of Water Quantity on Gastrointestinal (GI) Illnesses Outcomes.

Study	Setting and Participants	Gastrointestinal Illness Indicator	Water Quantity Measure(s)	Measure of Effect	Comparison Groups *	Values *	*p* Value
Esrey 1989 [26]	Lesotho; rural children (no age given)	Giardia lambia infection	Water usage	OR	8 lpcd	2.31 (1.25, 4.26)	<0.05
Jones 2007 [30]	Canada; rural and urban residents	Acute gastrointestinal illness (AGI) within past 28 days	Water consumption	OR	AGI *vs.* no AGI	1.06 (1.03, 1.09)	<0.05
Khan1982 [31]	Bangladesh; family contacts of Shigellosis cases	Shigellosis infection (among contacts of people receiving hand washing intervention)	Drinking and cooking water usage	OR	>5.5 L *vs.* < 4.5 L	0.51 (0.11, 3.78)	0.44
			Bathing and washing water usage	OR	≥25 L *vs.* <20 L	0.09 (0.003, 0.65)	0.008
		Shigellosis infection (among contacts of people receiving no hand washing intervention)	Drinking and cooking water usage	OR	>5.5 L *vs.* < 4.5 L	0.81 (0.33, 1.68)	0.58
			Bathing and washing water usage	OR	≥25 L *vs.* <20 L	1.70 (0.49, 7.83)	0.42
Srestha 2013 [38]	Nepal; all residents of rapidly urbanized HH	HH member experienced diarrhea in past month	Water consumption	OR	<20 lpcd	2.53 (1.10, 6.33)	not given
					20–49 lpcd	1.56 (0.63, 3.85)	
					50–99 lpcd	2.92 (1.17, 7.29	
					>100 lpcd	(ref)	
Teklemarium 2000 [39]	Ethiopia; rural children, <5 years	Child experienced diarrhea in past 2 weeks	Water consumption	OR	Diarrhea *vs.* no diarrhea	6.22 lpcd *vs.* 6.54 lpcd	>0.05
Tumwine 2002 [40]	Uganda, Tanzania, Kenya; rural and urban residents	HH member experienced diarrhea in past 7 days	Water usage	OR	Incremental increase of 1 lpcd	0.96 (0.93, 0.98)	0.001

* All comparisons are in units of water per day.

**Table 4:** Effects of Water Quantity on Growth Indicators.

Study	Setting and Participants	Growth Indicator	Water Quantity Measure(s)	Measure of Effect	Comparison Groups *	Values *	*p* Value
Esrey 1992 [27]	Lesotho; rural infants, 0–12 months	Weight gain (among families with a latrine)	Water usage	Difference	Increased usage *vs.* no increased usage	1.03 kg (0.42, 1.64)	<0.05
		Weight gain (among families without a latrine)		Difference	Increased usage *vs.* no increased usage	0.11 kg (−0.18, 0.39)	>0.05
		Length gain (among families with a latrine)		Difference	Increased usage *vs.* no increased usage	2.03 cm (0.53, 3.53)	<0.05
		Length gain (among families without a latrine)		Difference	Increased usage *vs.* no increased usage	−0.31 cm (−1.01, 0.39)	>0.05
Hebert 1985 [28]	India; rural children, 0–6 years	Weight-for-height	Washing water usage	Regression coefficient	0–18 months	0.60	0.50
					19–36 months	0.24	0.65
					36–72 months	0.25	0.37
			Cooking water usage	Regression coefficient	0–18 months	0.15	0.30
					19–36 months	0.07	0.32
					36–72 months	0.12	0.004
		Weight-for-age	Washing water usage	Regression coefficient	0–18 months	1.04	0.26
					19–36 months	0.26	0.70
					36–72 months	1.37	0.0003
			Cooking water usage	Regression coefficient	0–18 months	0.17	0.26
					19–36 months	0.09	0.29
					36–72 months	0.20	0.0003
		Height-for-age	Washing water usage	Regression coefficient	0–18 months	0.36	0.41
					19–36 months	0.08	0.83
					36–72 months	0.86	0.0003
			Cooking water usage	Regression coefficient	0–18 months	0.005	0.95
					19–36 months	0.04	0.35
					36–72 months	0.02	0.54
Zhang 2013 [43]	China; urban and rural primary and secondary students, 8–17 years	BMI	Water consumption	Comparison	Obese	8.94 L	<0.001
					Overweight	8.30 L	
					Normal	7.26 L	
					Underweight	6.81 L	
			Fluids consumption	Comparison	Obese	12.70 L	<0.001
					Overweight	12.02 L	
					Normal	10.67 L	
					Underweight	10.10 L	
			Beverage consumption	Comparison	Obese	3.76 L	<0.001
					Overweight	3.71 L	
					Normal	3.41 L	
					Underweight	3.29 L	

* All comparisons are in units of water per day.

**Table 5:** Effects of Water Quantity on Non-Communicable Disease (NCD) Outcomes.

Study	Setting and Participants	Non-Communicable Disease	Water Quantity Measure(s)	Measure of Effect	Comparison Groups *	Values *	*p* Value
Hu 2009 [29]	Canada; all residents	Renal cell carcinoma	Fluid consumption	OR	incremental increase of 0.3 L	1.04 (1.02–1.07)	0.0002
			Bottled water consumption	OR	none	(ref)	
					0–8 oz	0.95 (0.76, 1.18)	0.59
					>8 oz	0.95 (0.74, 1.22)	
			Tap water consumption	OR	<0.64 oz	(ref)	0.24
					0.64–20 oz	1.01 (0.85-1.19)	
					20–36 oz	1.10 (0.89, 1.37)	
					>36 oz	1.13 (0.88, 1.43)	
Pan 2012 [36]	United States; female nurses aged 25–42	Type II diabetes	Water consumption	RR	<1 c	0.93 (0.83, 1.05	0.15
					1 c	0.93 (0.83, 1.05)	
					2–3 c	1.09 (0.96, 1.24)	
					4–5 c	1.06 (0.91, 1.23)	
Vena 1993 [41]	United States; white male urban and rural residents, aged 35–90	Bladder cancer (among age <65)	Fluid consumption	OR	2–7 c	(ref)	<0.001
					8–10 c	2.60 (1.18, 5.73)	
					11–13 c	3.68 (1.65, 8.20)	
					14–49 c	6.30 (2.82, 14.08)	
			Tap water consumption	OR	0–5 c	(ref)	<0.001
					6–7 c	1.32 (0.72–2.42)	
					8–9 c	1.63 (0.90, 2.95)	
					10–39 c	2.62 (1.53, 4.47)	
		Bladder cancer (among age >65)	Fluid consumption	OR	2–7 c	(ref)	<0.001
					8–10 c	1.77 (1.08, 2.92)	
					11–13 c	1.80 (1.02, 3.19)	
					14–49 c	3.38 (1.83, 6.24)	
			Tap water consumption	OR	0–5 c	(ref)	<0.001
					6–7 c	1.28 (0.77, 2.14)	
					8–9 c	1.41 (0.81, 2.46)	
					10–39 c	2.98 (1.77, 5.03)	
		Bladder cancer (among never smokers)	Tap water consumption	OR	0–5 c	(ref)	not given
					6–7 c	4.17 (1.09, 15.96)	
					8–9 c	5.70 (1.46, 22.26)	
					10–39 c	25.51 (6.12, 106.29)	
		Bladder cancer (among ex-smokers)	Tap water consumption	OR	0–5 c	(ref)	not given
					6–7 c	0.82 (0.48, 1.41)	
					8–9 c	1.07 (0.61, 1.90)	
					10–39 c	1.61 (0.93, 2.78)	
		Bladder cancer (among current smokers, 1–28 pack years)	Tap water consumption	OR	0–5 c	(ref)	not given
					6–7 c	2.58 (0.49, 13.66)	
					8–9 c	2.70 (0.45, 16.13)	
					10–39 c	3.79 (0.77, 18.68)	
		Bladder cancer (among current smokers, >29 pack years)	Tap water consumption	OR	0–5 c	(ref)	not given
					6–7 c	1.87 (0.83, 4.22)	
					8–9 c	1.98 (0.89, 4.42)	
					10–39 c	3.56 (1.73, 7.31)	

* All comparisons are in units of water per day.

**Table 6:** Effects of Water Quantity on Mortality.

Study	Setting and Participants	Mortality Measure	Water Quantity Measure(s)	Measure of Effect	Comparison groups *	Values *	*p* Value
Aggarwal 2012 [23]	United States; urban and rural residents, >45 years	All-cause mortality	Water consumption	OR	none	1.93 (0.80, 4.63)	0.14
					0–2 c	1.44 (0.83, 2.50)	0.20
					2–4 c	0.75 (0.44, 1.28)	0.29
					4–6 c	1.27 (0.74, 2.17)	0.38
					6–8 c	(ref)	
					>8 c	1.22 (0.72, 2.07)	0.46
		Ischemia-related mortality	Water consumption	OR	none	2.79 (0.80, 9.80)	0.11
					0–2 c	1.81 (0.92, 3.52)	0.08
					2–4 c	1.41 (0.76, 2.63)	0.27
					4–6 c	1.74 (0.89, 3.39)	0.10
					6–8 c	(ref)	
					>8 c	1.01 (0.52, 1.95)	0.98
		Congestive heart failure-related mortality	Water consumption	OR	none	not given	
					0–2 c	1.93 (0.22, 16.95)	0.55
					2–4 c	1.12 (0.16, 7.69)	0.91
					4–6 c	0.96 (0.16, 5.85)	0.96
					6–8 c	(ref)	
					>8 c	0.33 (0.05, 2.42)	0.27
		Stroke-related mortality	Water consumption	OR	none	0.72 (0.14, 3.77)	0.69
					0–2 c	1.21 (0.33, 4.35)	0.77
					2–4 c	0.75 (0.24, 2.31)	0.61
					4–6 c	0.69 (0.22, 2.12)	0.52
					6–8 c	(ref)	
					>8 c	1.76 (0.42, 7.32)	0.44
Palmer 2012 [35]	Australia; urban residents	All-cause mortality	Water consumption	Hazard ratio (HR)	Incremental increase of 0.1 L	1.01 (0.99, 1.02)	>0.05
		Cardiovascular mortality	Water consumption	HR	Incremental increase of 0.1 L	1.05 (0.89, 1.12)	>0.05

* All comparisons are in units of water per day.

The results of these studies generally showed a positive association between higher levels of water consumption and higher growth indicators, but they also found important effect modifiers for this relationship. One study found a positive association between weight and height gain and higher levels of water usage, but only if the families owned a latrine [27]. Another study found a positive association between higher levels of water usage and growth indicators, but only among children over 36 months of age [28]. Finally, a study of school-aged children in China found a positive association between increased BMI and higher levels of water, fluid, and beverage consumption [43].

#### 3.3.4. Non-Communicable Diseases

Three studies, shown in Table 5, examined the relationship between water quantity and non-communicable diseases (NCDs). Two of the studies were case-control studies with a high risk of bias [29,41]; one of the studies was a prospective cohort study with a low risk of bias [36]. Two studies focused on adults only, with one further specifying an age range between 25 and 42 years old [36] and one further specifying an age range between 35 and 90 years old [41]. The third study provided no age restrictions on its population [29]. Higher levels of fluid consumption were found to be significantly associated with increased incidence of renal cell carcinoma and of bladder cancer, but they were not found to be significantly associated with Type II diabetes incidence [29,36,41]. The relationship with bladder cancer incidence was particularly strong among those under 65 years of age and among those who had never smoked tobacco [41].

#### 3.3.5. Mortality

Two studies, shown in Table 6, examined the effects of increased water consumption on all-cause and cardiac-related mortality. Both studies were prospective cohort studies, one with a high risk of bias [23] and one with a low risk of bias [35]. One study restricted its population to adults over the age of 45 years [23], and one provided no age restrictions on its population [35]. Neither study found a significant relationship between water consumption and either all-cause mortality or cardiac-related mortality [23,35].

## 4. Discussion

While water quantity is generally thought to be positively associated with health outcomes, this is mainly based on reviews of studies that rely on access as a proxy for the quantity of water in the home. This review, which was limited to studies that actually measured household water quantity, also found evidence of improved health from increased water quantity. However, the beneficial effect was largely dependent on how that water was used. Differences in study designs, settings, methods, and outcomes made a meta-analysis inappropriate, and the overall strength of the evidence was poor. Nevertheless, this review does provide some useful guidance on the relationship between water quantity and health.

### 4.1. Low- and Middle-Income Countries

For the communicable diseases examined—trachoma and GI-related illnesses—improved water quantity in the home often appeared to be significantly associated with improved disease outcomes, but in most cases this relationship depended on the manner in which the water was used.

For trachoma, which is a water-washed disease, simply bringing more water to the house or using more water for general household tasks was generally found not to be associated with improved trachoma indicators. Increased water that was used for face washing, by contrast, particularly face washing of children, was found to be significantly associated with lower prevalence of trachoma in the two studies that examined it.

Similarly, higher quantities of water in the home were generally associated with a lower odds of diarrheal disease. However, there was no evidence that increased consumption of water was protective. Rather, the evidence suggests that the health benefits were associated with increased use of water for personal and domestic hygiene.

For the growth indicators, higher levels of water consumption tended to be associated with higher weights and/or increased heights, but again only in certain groups. The finding that increased water quantity was only associated with increased growth in families that owned a latrine reinforces the importance of integrating increases in the quantity of water available with improvements in sanitation and hygiene.

Most included studies examining infectious diseases and growth outcomes in low and middle-income countries are cross-sectional studies, and many have serious methodological flaws that put these findings at a high risk of producing biased outputs. The relative consistency of the relationship of the findings and their accordance with the understood pathways of diseases add some credibility to the above summaries. Unfortunately, the described weaknesses in the included studies preclude any definitive statement of causality between water quantity and health outcomes; at best, these studies suggest an association between the two. In most of the included studies, it was also not made clear whether the groups using less water did so due to differences in preference (e.g., not wanting to wash the child’s face) or due to differences in availability of water (e.g., not having enough water to both wash the child’s face and provide adequate drinking water).

### 4.2. High-Income Countries

All of the studies that examined mortality and/or NCDs took place in high-income countries, namely the United States, Australia, and Canada. These studies were all either case-control or cohort studies, both of which generally produce more valid estimates than the cross-sectional studies more common in the included studies conducted in low- and middle-income countries. As noted in the risk of bias table, however, several of these studies had methodological flaws that increase the chance that bias affected their results. In particular, several studies had extremely low response rates and relied on recall periods of one to two years for self-reported water consumption.

For two of the three NCDs examined—renal cell carcinoma and bladder cancer—higher levels of water consumption were associated with increased odds of disease, while Type II diabetes incidence was not found to be associated with levels of water consumption. In both studies that examined mortality, neither all-cause mortality rates nor cardiac-related mortality rates were found to be associated with different levels of water consumption. Together, these findings suggest that increased levels of water consumption among residents of high-income countries might be associated with increased risk of a few specific NCDs, but these increased risks are not enough to significantly affect all-cause mortality rates.

### 4.3. Limitations and Further Research

This review identified several limitations in existing research on household water quantity that should be addressed by future studies. First, there is a paucity of studies that actually assess water quantity at the household level. While previous reviews have identified dozens of studies on the health impact of water quality and water access, few studies actually quantify the amount of water available. Even the proposed post-2015 targets simply rely on time to collect water as a proxy for water quantity [44]. The current available guidelines vary widely in their recommendations for the required amount of water per person per day, with a range of 15 to 50 L per capita per day [16,17,18,19,20,21]. Additional studies that directly measure the effects of changes in water quantity on health will allow for more valid, consistent, and evidence-based water quantity guidelines. Studies that examine water usage and the associated health outcomes in different settings, such as examining NCDs in low-income countries, would also be useful.

Second, the studies that do include a direct measurement of water quantity mainly follow a cross-sectional design with significant risks of confounding and bias. We identified no randomized controlled trials that assessed the impact of water quantity and health. In future, when researchers deliver interventions that are hypothesized to improve the quantity of water used by a household—such as building a standpipe in a household’s yard—they should measure the amount of water used by the household before and after the intervention and compare it to a valid counterfactual group. They could also measure the health impact of interventions that improve the efficiency of water use and so free up the quantity of water to be used for other tasks. In either case, if the intervention proves successful, the researchers could provide the counterfactual group with the intervention at the study’s close. These studies would allow for the conduct of randomized trials on water quantity without unethically restricting people’s water use, and they would also allow research on how people’s habits and preferences influence the quantity of water they use when restrictions on water quantity are eased. A supplementary source of evidence could be well-conducted, long-running surveys such as the Nurses’ Health Study cited in the section on NCDs, which could allow for much larger sample sizes than the randomized studies described above.

Third, the manner in which water quantity was actually measured varied significantly among studies and in some cases relied on self-reports whose accuracy has not been confirmed. There is a need for standard and validated approaches for measuring water quantity in the home, ideally using sensors or other technologies that are accurate and do not cause reactivity [45,46].

Finally, this review suggests that research to assess the impact of water quantity on health must also measure how the water is actually used—a gap in much of the existing research. People do not merely collect water; they use it for hygiene, drinking, cleaning, and other purposes, all of which provide different health benefits. Depending on the disease outcome of interest, studies should include measures of the amount of water used for consumption, for personal hygiene, and for cleaning, as these were identified in this review as being associated with at least one health outcome.

## 5. Conclusions

This review highlights the importance of measuring the quantity of water that people use for personal and household tasks. The available studies often focus on water quality rather than quantity, or they use access to water as a proxy for actual use. Future studies, particularly studies of interventions that are assumed to affect water quantity, should include direct measures of water quantity in order to enhance understanding of water quantity’s effects on health. Furthermore, simply measuring the amount of water brought to the household may not provide sufficient information on how that water is actually used. Researchers should also quantify how much water is used for tasks relevant to their health outcome of interest, such as water for face washing for trachoma prevention. With such improved data, international regulatory organizations would be able to provide more consistent evidence-based guidelines regarding water quantity requirements.

## Supplementary Files

Supplementary File 1
