# Supplementary material for: Household Water Quantity and Health: A Systematic Review
Source: Int J Environ Res Public Health. 2015 May 28;12(6):5954–74. doi: 10.3390/ijerph120605954 (PMC4483681; doi:10.3390/ijerph120605954)
Supplement: Supplementary File 1 [file ijerph-12-05954-s001.pdf]

Household Water Quantity and Health: A Systematic Review

Table S1. Detailed search strategy.

**_Concept 1: Interventions_**	
Subject headings	1. "drinking water"/exp
	2. "water supply"/exp
	3. "well water" /exp
	4. 1 or 2 or 3 (all MeSH terms)
Specific interventions	5. water adj2 (connect* or access* or require* or consum* or suppl* or distance*)
	6. (rainwater or "rain water") adj3 (collect* or harvest*)
	7. (standpipe* or handpump* or borehole*)
	8. 5 or 6 or 7
All interventions of interest	9. 4 or 8
Measures of water quantity	10. gallon* or quart* or pint* or cup* or ounce* or *liter*/ or *litre*/
	11. water adj3 (quantit* or amount* or volume* or much or usage)
	12. 10 or 11
Interventions related to water quantity	13. 9 and 12

Table S1. *Cont.*

Concept 2: Outcomes	
All health outcomes	14. diarrhea / exp
	15. morbidity or mortality or death*
	16. diarrhea* or diarrhoea*
	17. (water-borne or waterborne or water-wash*) adj3 disease*
	18. Cholera* or typhoid* or shigella* or dysentar* or cryptosporid* or giardia* or escherichia* or clostridium or rotavirus* or coli or norovirus* or campylobacter* or hepatitis
	19. "respiratory disease*"
	20. Dermatitis or folliculitis or lice or lymphatic filariasis or ringworm or tinea or scabies or trachom* or conjunctivitis or pinkeye or yaws or lepros* or typhus
	21. Injur* or Burn*
All health outcomes (continued)	22. malnutrition or malnourish* or undernutrition or undernourish* or nutrition* status
	23. anemi* or anaemi* or (vitamin adj3 deficienc*)
	24. stunt* or wast* or emaciat* or underweight
	25. BMI or body mass index
	26. Or/14-25
**Combined interventions and outcomes**	27. 13 and 26

© 2015 by the authors; licensee MDPI, Basel, Switzerland. This article is an open access article distributed under the terms and conditions of the Creative Commons Attribution license (http://creativecommons.org/licenses/by/4.0/).
